# Prognostic value of TMEM59L and its genomic and immunological characteristics in cancer

**DOI:** 10.3389/fimmu.2022.1054157

**Published:** 2022-12-23

**Authors:** Chang Shi, Lizhi Zhang, Dan Chen, Hong Wei, Wenjing Qi, Pengxin Zhang, Huiqi Guo, Lei Sun

**Affiliations:** ^1^ Department of Pathology and Forensic Medicine, College of Basic Medical Sciences, Dalian Medical University, Dalian, Liaoning, China; ^2^ Department of Pathology, First Affiliated Hospital, Dalian, China

**Keywords:** TMEM59L, pan-cancer, prognosis, tumor microenvironment, methylation

## Abstract

**Background:**

TMEM59L is a newly discovered transmembrane protein; its functions in cancer remain unknown. This study was designed to reveal the prognostic value and the functional role of TMEM59L in cancer.

**Methods:**

The gene expression profiles, methylation data, and corresponding clinical data of *TMEM59L* were retrieved from The Cancer Genome Atlas (TCGA) and the Genotype-Tissue Expression database. Survival analysis was employed to calculate the pan-cancer prognostic value of *TMEM59L*. The correlation between *TMEM59L* expression and tumor immune microenvironment, as well as DNA methylation dynamics and genomic heterogeneity across cancers were assessed based on data from TCGA.

**Results:**

Our findings revealed that distinct differences of *TMEM59L* mRNA expression were observed in different cancer types and that higher *TMEM59L* expression was observed in the advanced pathological stage and associated with worse prognosis in kidney renal papillary cell carcinoma, bladder urothelial carcinoma, colon adenocarcinoma, and kidney renal clear cell carcinoma. Pathway analysis indicated that *TMEM59L* exerted a key influence in cancer development and in immune- and cancer-associated pathways such as epithelial–mesenchymal transition and TGF-β signaling. Moreover, correlation analysis hinted at a negative correlation of *TMEM59L* expression with CD8 T cells, activated CD4 T cells, and several immunomodulators, including IDO1, TIGIT, PD-L1, CTLA-4, and BTLA in various cancers. Survival analysis indicated that the hypermethylation of *TMEM59L* gene was associated with longer survival times. A significant correlation was also observed between *TMEM59L* expression and immunophenoscore, homologous recombination deficiency, loss of heterozygosity, tumor stemness score, and neoantigens in various cancers. Importantly, we also identified numerous potential agents that may target *TMEM59L*.

**Conclusion:**

Our study revealed the prognostic value as well as the genomic and immunological characteristics of *TMEM59L* in cancers, highlighting the promising potential for TMEM59L as a prognostic cancer biomarker and a therapeutic target.

## 1 Introduction

The global incidence and mortality of cancer remain on the rise, with breast cancer, lung cancer, and colorectal cancer being the most common types of cancer with the highest mortality rates worldwide (1, 2). Cancer is a major cause of global mortality and a significant impediment to increasing life expectancy in the global population (3). Despite research efforts to improve cancer diagnosis and treatment, the associated clinical outcome and 5-year survival rate generally remain unfavorable, largely due to the complexity of this disease (4–8).

A large body of evidence has confirmed that the tumor microenvironment (TME) can determine abnormal tissue functions, alter the malignant behavior of tumor cells, and play vital roles in the consecutive evolution of malignant cancers and tumor resistance to anticancer drugs (9–11). The TME, characterized by hypoxia, oxidative stress, and abnormal levels of multiple cytokines and growth factors, induces dysplasia, which is defined as the emergence of heterogeneous tumor cell populations with distinct genetic and phenotypic characteristics (8, 12, 13). During cancer progression, tumor heterogeneity is exacerbated by the maturation of both cellular and acellular components of the TME (14, 15), enabling cancer stem cells (CSCs) to survive and proliferate – a principal attribute that underlies therapeutic resistance as well as tumor maintenance and recurrence (16–20). Multiple studies have indicated that genomic, epigenomic, and transcriptomic features are causally linked to the regulation of cancer pathways that support tumor cell growth and proliferation, and the phenomenon of cancer stemness (21–23). For these reasons, the outcome of current cancer chemotherapy, radiotherapy, and immunotherapy is far from satisfactory, and treatment regimens require further optimization.

DNA methylation signatures that are highly sensitive, specific, and analyzable have an enormous potential as clinical cancer biomarkers that play a non-negligible role in cancer diagnosis and prognosis, providing new technical means for early detection of different cancer types (24–27). Nevertheless, there is a need to explore new potential targets or cancer biomarkers to ensure that novel treatment regimens and appropriate combination therapy strategies can be specifically tailored to individual patients.

Transmembrane protein 59–like (TMEM59L), also known as brain-specific membrane-anchored protein BSMAP, was first discovered in 1999 (28). In 2006, using reverse transfection cell array technology, Mannherz et al. found that TMEM59L produced pro-apoptotic effects through an unknown mechanism (29). TMEM59L can regulate the N- and O-glycosylation steps that occur during Golgi maturation and is associated with glycosylation modifications of the amyloid precursor protein APP by inhibiting APP maturation, trafficking, and shedding (30). Recent studies have demonstrated that the downregulation of TMEM59L can protect neurons from oxidative stress, and that TMEM59L interacts with ATG5 and ATG16L1, partially activating LC3 and triggering autophagy (31, 32). Moreover, the homologue of TMEM59L, transmembrane protein 59 (TMEM59), is hypomethylated in late-onset Alzheimer’s disease, and methylation is involved in the transcriptional regulation and thus protein expression of TMEM59 (33). However, there is currently a lack of in-depth reports on the functional mechanism of TMEM59L, especially in the context of cancer research.

In this study, we comprehensively explored TMEM59L gene expression signature, its prognostic value, as well as its association with immune cell infiltration and cancer-associated pathways in various cancer types. Moreover, our study underscores the importance of TMEM59L as a prognostic biomarker and a treatment target and identified in TMEM59L a molecule to be further explored.

## 2 Materials and methods

### 2.1 Datasets

The gene expression profiles, methylation data, and corresponding pan-cancer clinical data were downloaded from The Cancer Genome Atlas (TCGA) database (https://portal.gdc.cancer.gov/), the Genotype-Tissue Expression (GTEx) dataset was downloaded from UCSC-hosted genomics platform (https://xenabrowser.net/). The cancer type abbreviations are listed in **Table 1**.

**Table 1:** The cancer type abbreviations are as above.

ACC	Adrenocortical carcinoma
BLCA	Bladder Urothelial Carcinoma
BRCA	Breast invasive carcinoma
CESC	Cervical squamous cell carcinoma and endocervical adenocarcinoma
CHOL	Cholangiocarcinoma
COAD	Colon adenocarcinoma
COADREAD	Colon adenocarcinoma/Rectum adenocarcinoma Esophageal carcinoma
ESCA	Esophageal carcinoma
GBM	Glioblastoma multiforme
GBMLGG	Glioma
HNSC	Head and Neck squamous cell carcinoma
KICH	Kidney Chromophobe
KIPAN	Pan-kidney cohort (KICH+KIRC+KIRP)
KIRC	Kidney renal clear cell carcinoma
KIRP	Kidney renal papillary cell carcinoma
LAML	Acute Myeloid Leukemia
LGG	Brain Lower Grade Glioma
LIHC	Liver hepatocellular carcinoma
LUAD	Lung adenocarcinoma
LUSC	Lung squamous cell carcinoma
OV	Ovarian serous cystadenocarcinoma
PAAD	Pancreatic adenocarcinoma
PCPG	Pheochromocytoma and Paraganglioma
PRAD	Prostate adenocarcinoma
READ	Rectum adenocarcinoma
SARC	Sarcoma
STAD	Stomach adenocarcinoma
SKCM	Skin Cutaneous Melanoma
STES	Stomach and Esophageal carcinoma
TGCT	Testicular Germ Cell Tumors
THCA	Thyroid carcinoma
UCEC	Uterine Corpus Endometrial Carcinoma

### 2.2 Integrated network and enrichment analysis

Each patient was divided into a high-expression or a low-expression group based on the median of *TMEM59L* expression. We used the GSVA R package to conduct the gene set enrichment analysis (GSEA) to evaluate pathway enrichment for high- and low-*TMEM59L* expression groups (34). Hallmark gene sets (h.all.v7.2.symbols) were collected from GSEA database (http://www.gsea-msigdb.org/gsea/downloads.jsp). Reverse phase protein array (RPPA) data from TCPA database (https://www.tcpaportal.org/tcpa/index.html) were also used to assess pathway activity score (PAS). The evaluated pathways included apoptosis, cell cycle, DNA damage response, epithelial–mesenchymal transition (EMT), as well as hormone androgen receptor (AR), hormone estrogen receptor (ER), tuberous sclerosis complex–mammalian target of rapamycin (TSC–mTOR), receptor tyrosine kinase (RTK), Ras/MAPK (mitogen-activated protein kinase), and PI3K/AKT signaling pathways, all of which are notably associated with cancer. The difference of PAS was evaluated using Student’s *t*-test, and the resulting *p*-value was adjusted for false discovery rate (FDR), with FDR ≤ 0.05 being considered significant. When PAS (TMEM59L High expression) > PAS (TMEM59L Low expression), we considered TMEM59L to have an activating effect on a specific pathway; in the opposite case TMEM59L was considered to have an inhibitory effect on a pathway.

### 2.3 Estimation of immune cell infiltration

The correlation of *TMEM59L* expression with the immune infiltration level was assessed using the CIBERSORT algorithm (https://cibersort.stanford.edu) (35). The stromal, immune, and ESTIMATE scores for each patient were calculated using the ESTIMATE algorithm (36). The immunophenoscore (IPS) for each patient was calculated according to the method reported by Charoentong (37). We also extracted the expression data of 155 immunomodulators including chemokines, receptors, MHC, immune-inhibitors, and immune-stimulators from each patient based on the study of Charoentong et al. (37) as well, and correlation analyses were subsequently conducted to assess the association between immunological characteristics and *TMEM59L* across cancer types.

### 2.4 Methylation analysis

We downloaded the methylation data from TCGA database. In total, 14 cancer types were selected and analyzed including Colon adenocarcinoma (COAD), Colorectal carcinoma (COADREAD), Thyroid carcinoma (THCA), Cholangiocarcinoma (CHOL), Liver hepatocellular carcinoma (LIHC), Kidney renal papillary cell carcinoma (KIRP), Pan-kidney cohort (KIPAN), Adrenocortical carcinoma (ACC), Ovarian serous cystadenocarcinoma (OV), Uterine Corpus Endometrial Carcinoma (UCEC), Rectum adenocarcinoma (READ), Stomach and Esophageal carcinoma (STES), Breast invasive carcinoma (BRCA), Bladder Urothelial Carcinoma (BLCA), Kidney renal clear cell carcinoma (KIRC), Prostate adenocarcinoma (PRAD), Stomach adenocarcinoma (STAD), Lung squamous cell carcinoma (LUSC), Lung adenocarcinoma (LUAD), Pancreatic adenocarcinoma (PAAD), Glioma (GBMLGG), Esophageal carcinoma (ESCA), Kidney Chromophobe (KICH), and Head and Neck squamous cell carcinoma (HNSC). The cohort included more than 10 paired cancer and adjacent non-cancer samples. Spearman correlation analyses were performed to identify whether *TMEM59L* expression was associated with methylation levels.

### 2.5 Drug analysis

We recorded the drug sensitivity data from Genomics of Drug Sensitivity in Cancer (GDSC) database (38) and the Genomics of Therapeutics Response Portal (CTRP) database (39). Spearman correlation analysis was carried out to identify the association between gene mRNA expression and drug response.

### 2.6 Statistical analysis

We computed the statistical analyses in the R (version 4.1.1). Hazard analyses were carried out using Cox regression. Survival curves were analyzed by log-rank test. Correlation coefficients were obtained using the Spearman correlation method. Any *p*-value less than 0.05 was considered statistically significant.

## 3 Results

### 3.1 *TMEM59L* mRNA expression in human cancers

**Figure 1:**
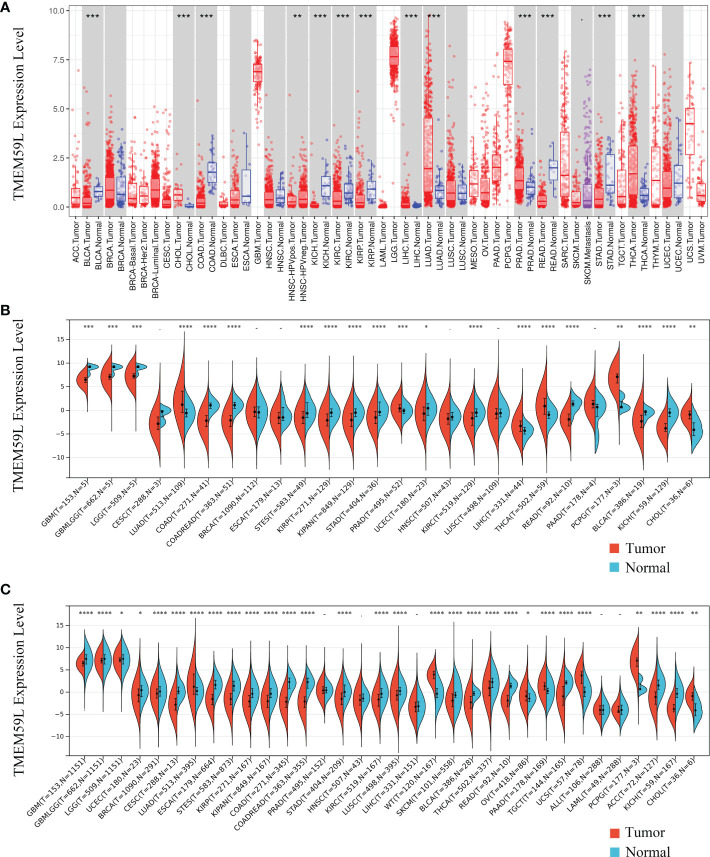
TMEM59L mRNA expression in different types of human cancers. **(A)** TMEM59L mRNA expression in different tumor types compared with normal tissues in the TIMER database. **(B)** TMEM59L mRNA expression in different tumor types compared with normal tissues from TCGA database. **(C)** mRNA expression of TMEM59L across tumor types using TCGA and GTEx data. (*P < 0.05, **P < 0.01, ***P < 0.001), ****p < 0.0001.

The TIMER online database (https://cistrome.shinyapps.io/timer/) was first used to identify the expression of *TMEM59L* mRNA transcripts in different types of cancer (**Figure 1A**). Compared with corresponding normal tissues, *TMEM59L* mRNA expression was significantly increased in six human cancers, specifically BRCA, CHOL, LIHC, LUAD, PRAD, and THCA. In contrast, *TMEM59L* expression was evidently lower in BLCA, COAD, KICH, KIRC, KIRP, and STAD than that in the normal tissues. Subsequently, a pan-cancer analysis demonstrated that *TMEM59L* expression was decreased across most cancer types, such as GBM, GBMLGG, KIRP, COAD, KICH, KIRC, LGG, KIPAN, COADREAD, STAD, UCEC, READ, STES, and BLCA (**Figure 1B**). Considering the small number of normal samples in TCGA database, we integrated the data of normal tissues from the GTEx database with the data of TCGA tumor tissues to determine the expression characteristics of TMEM59L across the pan-cancer cohort. The results were similar; compared with its expression in normal samples, *TMEM59L* was significantly downregulated in most cancer types (**Figure 1C**).

### 3.2 *TMEM59L* expression profile at different clinical stages or in different cancer subtypes

**Figure 2:**
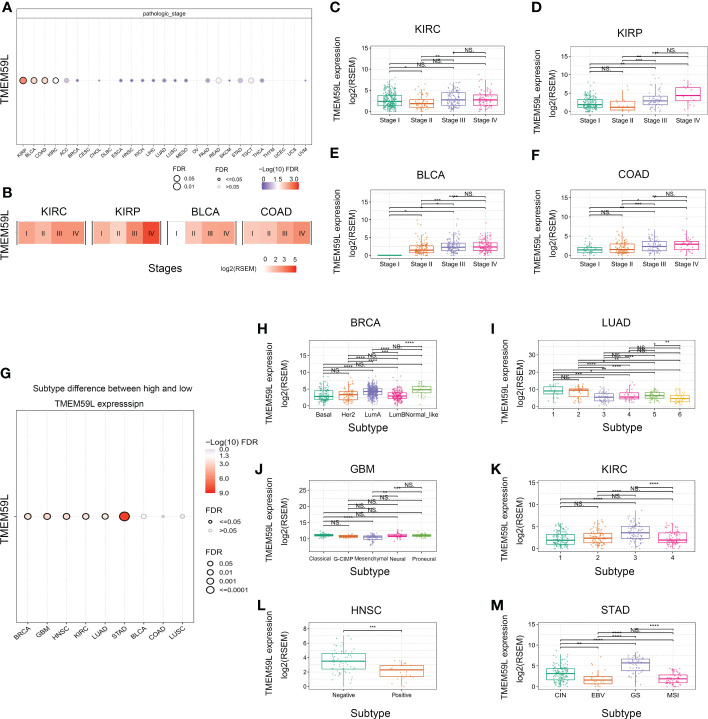
TMEM59L expression at different clinical stages or subtypes of different cancers. **(A)** The difference of TMEM59L mRNA expression between pathologic stages in the specific cancers. **(B)** Heatmap presents the TMEM59L mRNA expression profile among stages in the specific cancers. **(C–F)** TMEM59L mRNA expression in pathologic stage of KIRC, KIRP, BLCA, and COAD. **(G)** The associations between subtypes and TMEM59L expression. **(H–M)** TMEM59L mRNA expression in subtypes of BRCA, LUAD, GBM, KIRC, HNSC, and STAD. (ns: not significant, *P < 0.05, **P < 0.01, ***P < 0.001, ****p < 0.0001).

We further analyzed *TMEM59L* mRNA expression tendency at different clinical stages and in different cancer subtypes (**Figure 2A**). Distinct differences could be observed in varying clinical stages in several cancer types, including KIRP, BLCA, COAD, and KIRC. Remarkably, in KIRP, BLCA, COAD, and KIRC, later pathological stage showed higher *TMEM59L* mRNA expression (**Figures 2B–F**). Furthermore, *TMEM59L* mRNA expression in LUAD, GBM, HNSC, BRCA, KIRC, and STAD was also significantly different based on the molecular specific subtype (**Figures 2G–M**). To increase the reliability of our study, we verified the protein expression level of TMEM59L. Based on the HPA database (https://www.proteinatlas.org/), we further explored the protein level of TMEM59L in normal tissues and human cancers. **Figure S1A** showed the protein expression level of TMEM59L in normal tissues. The immunohistochemical results showed that the expression level of TMEM59L is not high in most tissues except for the pituitary gland; Subsequently, we also explored the expression of TMEM59L in cancer tissues. As shown in **Figure S1B**, TMEM59L has a relatively high protein expression level in colorectal cancer, pancreatic cancer, kidney cancer, and liver cancer. These results were consistent with our previous results that the later the stage, the higher mRNA level of TMEM59L in COAD and KIRP. **Figure S1C** further showed the representative IHC images of TMEM59L in colorectal and renal cancer based on HPA database.

### 3.3 Prognostic value of *TMEM59L* mRNA expression

**Figure 3:**
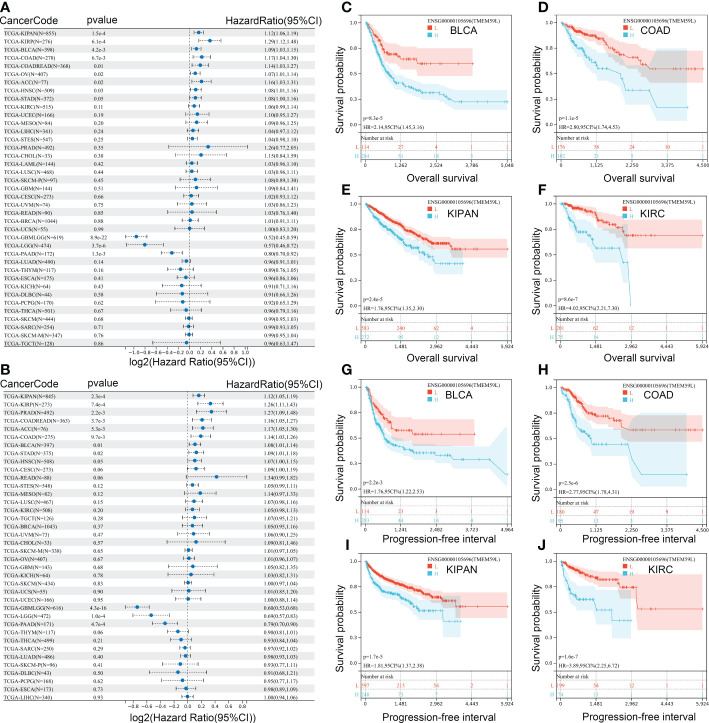
Correlation analysis between mRNA expression of TMEM59L and prognostic value. **(A)** The Overall survival (OS) difference between high and low TMEM59L expression groups. **(B)** The Progression-free interval (PFI) difference between high and low TMEM59L expression groups. **(C–J)** OS and PFI difference between high and low TMEM59L expression groups in BLCA, COAD, KIPAN, and KIRC.

To further identify the prognostic value of *TMEM59L*, we then performed a survival analysis on the data retrieved from the TCGA database. Cox regression indicated that a high *TMEM59L* expression was associated with shorter overall survival (OS) and progression-free interval (PFI) of KIPAN, KIRP, BLCA, COAD, COADREAD, OV, ACC, HNSC, and STAD (**Figures 3A, B**). In contrast, higher *TMEM59L* expression predicted longer OS and PFI in GBMLGG, LGG, and PAAD (**Figures 3A, B**). Further survival curves also indicated that high *TMEM59L* expression was associated with worse OS (**Figures 3C–F**) and PFI in BLCA, COAD, KIRC, and KIRP (**Figures 3G–J**). Meanwhile, there was no significant association between *TMEM59L* expression and clinical outcome in other cancers.

### 3.4 Association between *TMEM59L* mRNA expression and cancer-related pathways

**Figure 4:**
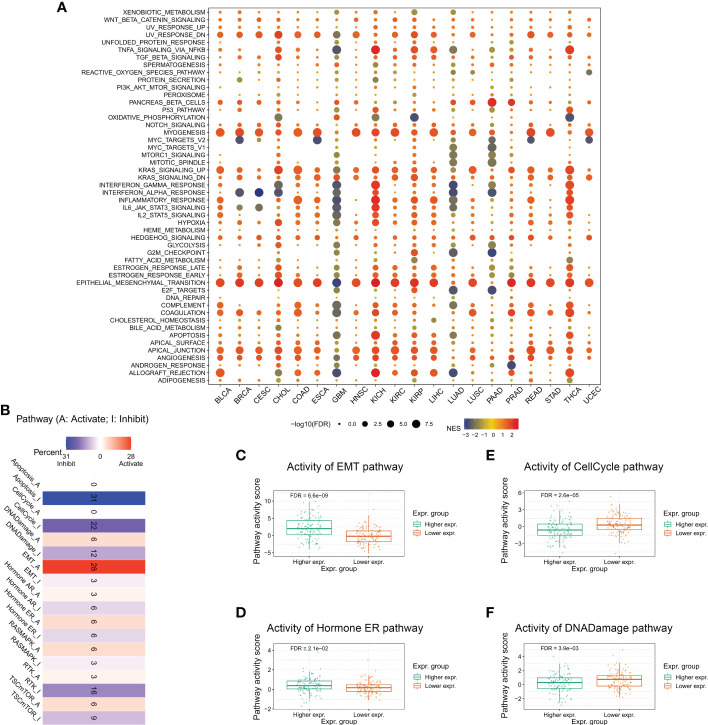
Association between TMEM59L and pathways in cancers. **(A)** Enrichment analysis for cancer signaling between high and low TMEM59L expression tumor tissues. NES is the normalized enrichment score in the GSEA algorithm. **(B)** The combined percentage of the effect of TMEM59L on pathway activity indifferent types of human cancers. **(C–F)** The differences of epithelial mesenchymal transition (EMT), Cell Cycle, Hormone estrogen receptor (ER), and DNA damage pathways activity between high and low TMEM59L expression groups in COAD.

To better understand the relevance and potential functions of *TMEM59L* in cancer pathogenesis, we performed functional enrichment analysis on the low and high *TMEM59L* expression groups across several cancer types (**Figure 4A**). The results indicated that *TMEM59L* expression was closely correlated with cancer-related hallmarks, including epithelial-mesenchymal transition (EMT), P53 pathway, E2F target, cell cycle regulation at G2-M, KRAS signaling, WNT beta-catenin signaling, and immune-related pathways, such as TGF-β, IL2-STAT5, and TNFα signaling *via* NF-kB. Moreover, the pathway activity analysis suggested that *TMEM59L* was significantly involved in 10 salient cancer-related pathways, namely DNA damage response, apoptosis, RTK, cell cycle, Hormone AR, Hormone ER, TSC–mTOR, Ras/MAPK, EMT and PI3K/AKT signaling pathways (**Figure 4B**). The main pathway activated by *TMEM59L* was EMT (28% activation vs. 3% inhibition), especially in BLCA, BRCA, COAD, ESCA, OV, READ, STAD, TGCT, and THCA (**Figure S2**), whereas the pathways inhibited by *TMEM59L* included apoptosis (31% inhibition vs. 0% activation) and cell cycle (22% inhibition vs. 0% activation). When compared with low TMEM59L expression group, the activities of EMT and estrogen receptor (ER) pathways were also higher, whereas a lower pathway activity in cell cycle and DNA damage response was observed in the high *TMEM59L* expression group for patients with COAD (**Figures 4C–F**). The above results suggested that *TMEM59L* exerts a key influence on cancer pathogenesis and development.

### 3.5 Interaction network of *TMEM59L*

**Figure 5:**
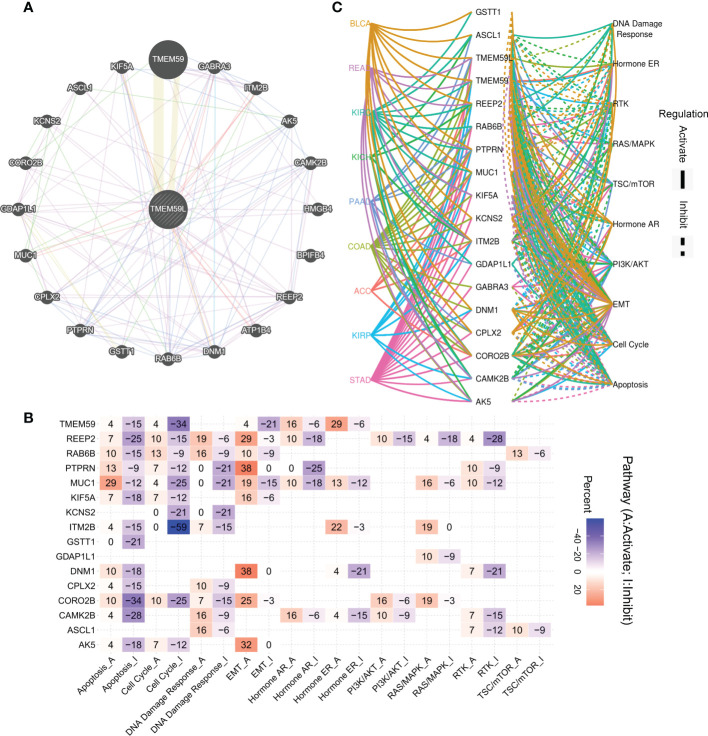
Association between interaction genes of TMEM59L and pathways in cancers. **(A)** Interaction Network of TMEM59L constructed by GeneMANIA. **(B)** The combined percentage of the effect of interaction genes of TMEM59L on pathway activity in different types of human cancers, the number in each cell means that the percentage of cancer types, in which TMEM59L showed significant association with the specific pathway, among the selected cancer types. **(C)** Association between interaction genes of TMEM59L and known pathways in ACC, BLCA, COAD, READ, STAD, KIRP, KIRC, KICH and PAAD. (solid line: activation; dashed line: inhibition), the different colors of the lines represent different types of cancer.

Based on the GeneMANIA database, the 20 proteins most closely correlated with TMEM59L expression, namely TMEM59, GABRA3, ITM2B, AK5, CAMK2B, HMGB4, BPIFB4, REEP2, ATP1B4, DNM1, RAB6B, GSTT1, PTPRN, CPLX2, MUC1, GDAP1L1, CORO2B, KCNS2, ASCL1, and KIF5A, were analyzed to construct a protein-protein interaction network (**Figure 5A**). Subsequently, these interacting genes were subjected to functional enrichment analysis, and consistently with the previous results, these genes were significantly enriched in the activation of EMT signaling pathway and in the inhibition of apoptosis and cell cycle signaling pathway (**Figure 5B**). Relative network analysis also indicated that *TMEM59L* and its interacting genes were involved in cancer-related pathways, such as TSC/mTOR, RTK, EMT, Ras/MAPK, and PI3K/AKT signaling, particularly in ACC, BLCA, COAD, READ, STAD, KIRP, KIRC, KICH, and PAAD (**Figure 5C**).

#### 3.5.1 Association of *TMEM59L* expression with the tumor immune microenvironment

**Figure 6:**
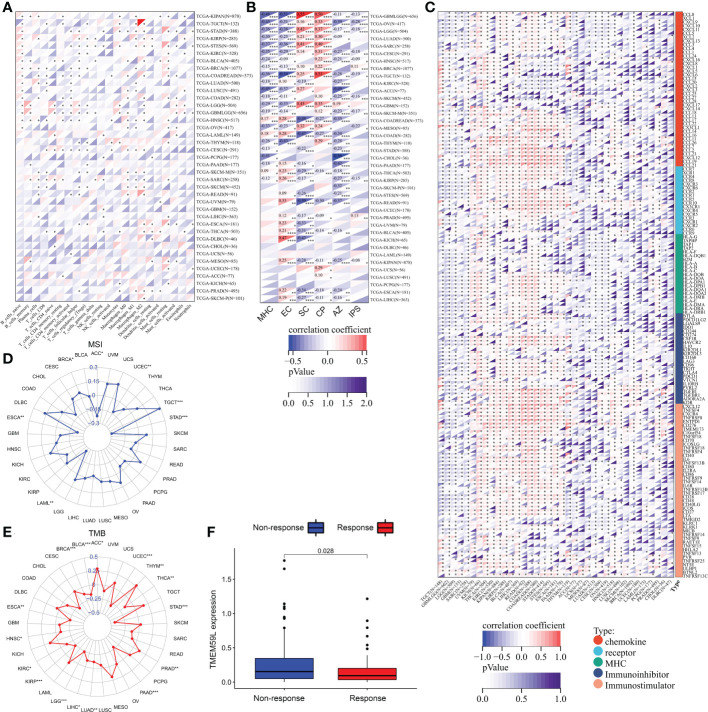
Relationship between TMEM59L expression and the tumor-immune microenvironment. **(A)** The correlation of TMEM59L expression with immune cell infiltration levels in pan-cancer. **(B)** The correlation between TMEM59L expression and the Immunophenoscore (IPS) across various cancer types. CP, immune checkpoints; SC, suppressor cells; EC, Effector cells; AZ, Average Z-score. **(C)** Correlation between TMEM59L and 155 immunomodulators, including chemokine, receptor, MHC, immuno-inhibitor, and immuno-stimulator across cancers. **(D, E)** Correlation of TMEM59L expression with tumor mutation burden (TMB) and microsatellite instability (MSI) in multiple cancer. **(F)** Patients with high TMEM59L expression have a worse clinical response to immune therapy in IMvigor210 cohort. (*p < 0.05, **p < 0.01, ***p < 0.001, ****p < 0.0001).

As the pathway enrichment analysis revealed that *TMEM59L* was closely related to inflammation and immune function, we further investigated the link between *TMEM59L* expression and immune cell infiltration levels using the CIBERSORT algorithm. The results demonstrated that *TMEM59L* expression was distinctly negatively correlated with immune infiltration levels in LUSC, SARC, COADREAD, LUAD, HNSC, CESC, BRCA, and TGCT, especially with the levels of CD8 T cell and activated CD4 T cells (**Figure 6A** and **Table S1**). We then further assessed Spearman’s correlation coefficient of *TMEM59L* and immune scores across distinct cancer types using the ESTIMATE algorithm. A significantly positive correlation between *TMEM59L* and stromal scores was detected, yet a negative correlation with immune scores across many cancer types (**Table S2**). IPS has been shown to effectively predict the response rate to anti-CTLA-4 and anti-PD-1 therapy. For this reason, we investigated the link between *TMEM59L* expression and the IPS across various cancer types. **Figure 6B** showed that *TMEM59L* expression was evidently negatively correlated with IPS in several types of cancers, including GBMLGG, LGG, OV, CESC, KIRC, SKCM, KIRP, and KIPAN. Moreover, IPS analysis demonstrated that *TMEM59L* expression was positively associated with immune checkpoints (CP) and suppressor cells (SCs) but was negatively correlated with MHC, average Z-score (AZ), and effector cells (ECs) in most tumors, all the p-values are less than 0.05.

We also demonstrated that *TMEM59L* expression was negatively linked with the expression of many immune modulators, including PD-L1, IDO1, TIGIT, CTLA-4, and BTLA in various cancers (**Figure 6C**). *TMEM59L* also showed a negative correlation with tumor mutational burden (TMB) in many cancers, such as HNSC, LUAD, LIHC, KIRC, BRCA, THCA, BLCA, KIRP, LGG, ESCA, PAAD, UCEC, and STAD and a negative correlation with microsatellite instability (MSI) in UCEC, ACC, ESCA, LAML, and STAD, which suggest that *TMEM59L* may reflect cancer immunogenicity in these cancer types (**Figures 6D–E** and **Table S3**). Subsequently, based on the IMvigor210 cohort, we also found a link between the high expression of *TMEM59L* and poor clinical response to immune therapy (**Figure 6F**). These observations may hint at an intricate interplay between *TMEM59L* and the immune microenvironment, although more in-depth investigations are needed to unveil the specific molecular mechanisms.

To further clarify the possible role of TMEM59L in the tumor microenvironment, we analyzed single-cell sequencing data from BRCA-GSE148673 dataset through the TISCH database (a scRNA-seq database that provides extensive cell type annotations at the single-cell level, allowing TME exploration across various cancers). The results of UMAP showed that 28 clusters were identified in the BRCA-GSE148673 dataset (**Figure S3A**), and then the corresponding clusters were labeled into nine different cell subpopulations, including B cell, CD4 T conv, CD8 T cell, endothelial, epithelial, fibroblasts, malignant, mono/macro, and Tprolif (**Figure S3B**). For the BRCA-GSE148673 data set, TMEM59L is mainly expressed in fibroblasts (**Figures S3C, D**). Previous studies have shown that fibroblasts are mainly involved in the activation of the EMT pathway to promote metastasis (40–42), and functional enrichment analysis subsequently conducted further confirmed our speculation that the activity of the EMT and angiogenesis pathways in TMEM59L high-expressing cell cluster (fibroblasts) was significantly increased (**Figures S3E, F**). All the above results indicated that TMEM59L participates in tumor invasion and metastasis through the activity EMT pathway, which was consistent with our previous results.

We also performed GSEA analysis using TCGA-BRCA bulk RNA-seq data to compare the expression level of TMEM59L concerning related signaling pathways. The cancer-associated pathway signatures were extracted from Jiao Hu et al. (43), the cancer-immunity cycle reflects the anticancer immune response (44), and the activation levels cancer-immunity cycle were retrieved from tracking tumor immunophenotype (TIP) (45)(http://biocc.hrbmu.edu.cn/TIP/). And as shown, TMEM59L was significantly positively correlated with oncogenic pathways (such as Ta_pathway, EMT_differentiation, and Myofibroblasts pathway) (**Figure S4A**). Interestingly, we further found that TMEM59L is negatively correlated with cancer immunity cycle pathways which further confirmed that TMEM59L is related to the immunosuppressive microenvironment (**Figure S4B**).

### 3.6 DNA methylation alterations across *TMEM59L* gene across different human cancers

**Figure 7:**
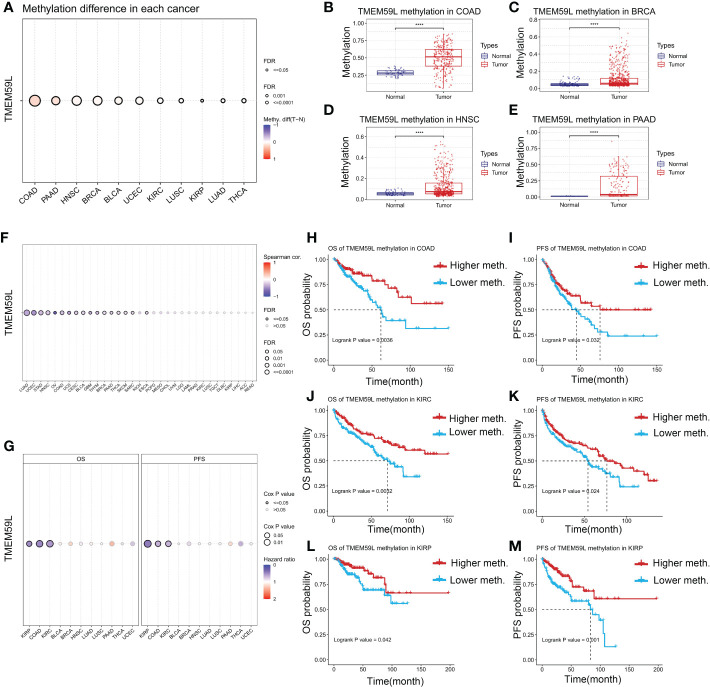
DNA methylation alterations of TMEM59L across different human cancers. **(A)** The methylation difference between tumor and normal samples of TMEM59L in different human cancers. **(B–E)** TMEM59L methylation in COAD, BRCA, HNSC, and PAAD. **(F)** The correlation between methylation and mRNA expression of TMEM59L in different human cancers. **(G)** The OS and PFS difference between higher and lower TMEM59L methylation groups in different human cancers. **(H–M)** The prognosis analysis of TMEM59L methylation in COAD, KIRC and KIRP.

Epigenetic changes such as DNA methylation play key roles in modulating the behaviors of cancer cells and immune tolerance (46), thus we explored whether epigenetic regulation is involved in *TMEM59L* mRNA expression. As shown in **Figure 6A**, the methylation levels of *TMEM59L* gene in distinct cancers were highly heterogeneous (**Figure 7A**). The *TMEM59L* gene was hypermethylated in most cancers, including COAD (**Figure 7B**), BRCA (**Figure 7C**), PAAD (**Figure 7D**), HNSC (**Figure 7E**), BLCA, UCEC, KIRC, and LUSC, whereas it was hypomethylated in KIRP, LUAD, and THCA (P < 0.05, **Figure S5**). Spearman correlation analysis indicated that *TMEM59L* expression correlated negatively with its gene methylation level in BLCA, BRCA, COAD, UCEC, HNSC, LUAD, PAAD, and THCA (FDR < 0.05; **Figure 7F** and **Figure S6**). Subsequently, survival analysis also showed that the hypermethylation of the *TMEM59L* gene correlated with longer survival times than the survival times associated with the hypomethylation of *TMEM59L* gene (P < 0.05, **Figure 7G**), especially in COAD, KIRC, and KIRP. The hypermethylation of *TMEM59L* was significantly correlated with longer OS and PFI (**Figures 7H–M**). No association was found between *TMEM59L* methylation and survival in other cancer types.

### 3.7 Correlation analysis of *TMEM59L* expression with stemness index and genomic heterogeneity across cancers

**Figure 8:**
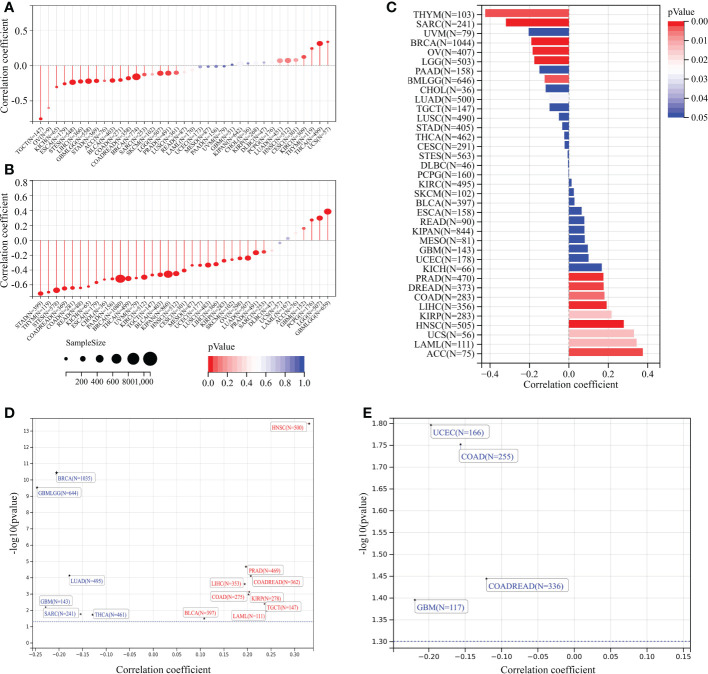
Correlation analysis of TMEM59L expression with stemness index and genomic heterogeneity across cancers. **(A, B)** The association between TMEM59L expression and tumor stemness score (DNAss and RNAss) in different cancers. **(C)** The association between TMEM59L expression and the homologous recombination deficiency (HRD) in different types of cancer. **(D, E)** The correlation of TMEM59L expression with heterozygosity (LOH) and Neoantigens (NEO) in different types of cancer.

Stem cell–like characteristics have been established as the main cause of chemoresistance (47, 48) and the key drivers of tumor progression (49–51). In the present study, we conducted correlation analyses to identify the association between *TMEM59L* expression and tumor stemness scores (RNA and DNA stemness scores). A significant negative correlation between DNA stemness score and *TMEM59L* expression in most tumors was observed in LGG, ESCA, SARC, STES, GBMLGG, STAD, COAD, LIHC, BRCA, TGCT, COADREAD, BLCA, PRAD, and KICH (**Figure 8A**). Similar results were seen when assessing the correlation between RNA stemness score and *TMEM59L* expression in most cancers, except for GBM, GBMLGG, LGG, and PCPG (**Figure 8B**). Homologous recombination is a critical pathway for double-strand break repairs (52, 53), thus homologous recombination deficiency would result in a high level of genomic instability, leading to a loss of heterozygosity and ultimately cell death (52, 54). Homologous recombination deficiency cancers have been shown to be markedly correlated with sensitivity to platinum-based chemotherapeutic drugs and PARP inhibitors (55, 56). In the current study, the expression of *TMEM59L* was closely related to homologous recombination deficiency status in most tumors (**Figure 8C**), and further loss of heterozygosity analysis showed a significantly positive association between loss of heterozygosity status and *TMEM59L* expression in several cancers, such as COAD, COADREAD, LAML, KIRP, PRAD, HNSC, LIHC, TGCT, and BLCA but a negative association with GBM, GBMLGG, LUAD, BRCA, SARC, and THCA (**Figure 8D**). Neoantigens were reported to be critical targets of immunotherapy and were correlated with improved clinical outcome and response rate to immune checkpoint blockade in several cancers, such as non-small cell lung cancer and melanoma (57–61). Our study discovered that *TMEM59L* expression was linked with neoantigens in only a limited number of cancers, such as COAD, COADREAD, GBM, UCEC, while no link was evident in other cancers (**Figure 8E**).

### 3.8 Drug sensitivity analysis

**Figure 9:**
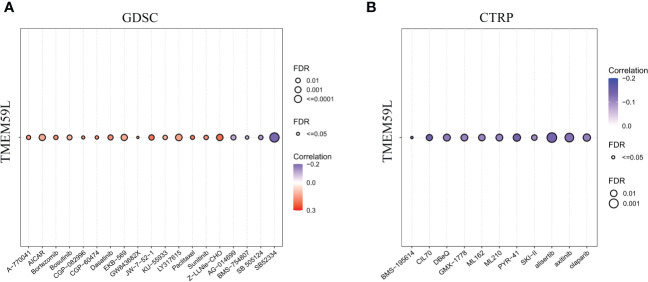
Drug sensitivity analysis. **(A)** The correlation between TMEM59L expression and the sensitivity of GDSC drugs in pan-cancer. **(B)** The correlation between TMEM59L expression and the sensitivity of CTRP drugs in pan-cancer. Blue bubbles represent negative correlations, red bubbles represent positive correlations, the deeper of color, the higher of the correlation. Bubble size is positively correlate with the FDR significance. Black outline border indicates FDR ≤ 0.05.

Genomic aberrations would impact the sensitivity of malignant tumors to drug therapy (including chemotherapy and targeted therapy) (62). Since *TMEM59L* expression was closely associated with the genomic heterogeneity of various cancers, we then performed the drug sensitivity analysis on the GDSC (38) and CTRP databases. The results indicated that patients with high *TMEM59L* expression were more susceptible to AG-01469, BMS-754807, SB 505124, CIL70, DBeQ, ML162, ML210, axitinib, alisertib, olaparib, PYR-41, GMX-1778, BMS-195614, and B52334 (negative correlation with IC50, *p* < 0.05; **Figures 9A, B**). This implied that the dysregulation of *TMEM59L* could lead to anti-tumor drug resistance.

## 4 Discussion

Transmembrane proteins (TMEMs) are proteins that span the entirety of the cell membranes (63), and many of such proteins play an important role in cancer development and cancer cell dissemination (64, 65), by mediating signal transduction between the cytoplasmic proteins and extracellular environment (66). Therefore, TMEMs represent attractive drug targets for cancer therapy (64). TMEM59L is a newly discovered brain-specific membrane-anchored protein that has been reported to act as a pro-apoptotic protein (29, 31). TMEM59L downregulation protects neurons from oxidative stress (31). Recent studies have also shown that *TMEM59L* can also regulate autophagy-related biological processes (32). However, there is currently a dearth of systematic studies in the literature on the TMEM59L regulation of tumor pathophysiology across cancer types.

In the present research, we assessed the pan-cancer expression of *TMEM59L* and the correlation of dysregulation of *TMEM59L* expression with clinical outcome of patients. The results indicated that *TMEM59L* expression was altered in different types of cancer and associated with the clinical outcome of cancer patients. *TMEM59L* expression was evidently downregulated across most cancer types compared to its expression in the corresponding normal tissues. Further analysis demonstrated that distinct differences was observed in different clinical stages of several cancer types, such as KIRP, BLCA, COAD, and KIRC, where advanced tumor stage correlated with higher *TMEM59L* mRNA expression. Therefore, in these specific cancer types *TMEM59L* may serve as a tumor promoting factor. Additionally, survival analysis confirmed that *TMEM59L* was a risk factor in patients with KIRP, BLCA, COAD, and KIPAN (KIRC+KIRP+KICH).

The mechanism by which *TMEM59L* regulates tumorigenesis and cancer pathophysiology remains unclear, but the relationship we observed between *TMEM59L* and the hallmarks of cancer could improve our understanding of the functional roles of *TMEM59L*. GSEA analysis demonstrated that *TMEM59L* expression was strictly linked with hallmarks of malignancy and immune-related pathways in most cancers, such as EMT, P53, apoptosis, cell cycle, WNT, IL-6-JAK-STAT3, IL2-STAT5 and TGF-β signaling pathways.

Genetic and epigenetic changes play key roles in immune tolerance and cancer development (46). In our study, the abnormal hypermethylation of *TMEM59L* was associated with decreased mRNA levels and better clinical outcomes for several cancers, such as KIRP, KIRC, and COAD, suggesting that hypermethylation of *TMEM59L* gene may be key regulatory mechanism for *TMEM59L* expression in these cancers. Interestingly, in line with our previous findings, high *TMEM59L* expression were associated with poor prognosis in COAD, KIRC, and KIRP. Thus, we speculated that the epigenetic changes of *TMEM59L* gene may promote the occurrence of KIRC, KIRP, and COAD in some cases.

Tumor immunotherapy has made remarkable achievements in cancer treatment (67). Immune checkpoint blockade therapy has significantly prolonged the survival in many cancers typically associated with poor prognosis, such as melanoma and non-small cell lung cancer (68). However, immunotherapy is still only available for a subset of patients, and immunotherapy response rates vary widely across cancer types (69, 70). Our study found that in addition to regulating pathways involved in cancer progression, *TMEM59L* was also involved in immune regulatory pathways such as IL6-JAK-STAT3, IL2-STAT5, and TGF-β signaling. Correlation analysis showed that *TMEM59L* expression negatively correlated with activated CD4 T cells and CD8 T cells in most cancer types, and further IPS analysis also replicated the same trend; *TMEM59L* expression was negatively related to IPS score, AZ, and ECs, while being positively associated with SCs, indicating that TMEM59L could play a key role in the immunosuppressive microenvironment. At the same time, the close association of TMEM59L with most immunomodulators and immune checkpoints also implied that TMEM59L could predict the clinical response of patients to immune checkpoint blockade, and this was validated in the IMvigor210 cohort, as high expression of *TMEM59L* correlated with a worse clinical response to PD-L1 therapy. Taken together, all of the results presented above suggested that *TMEM59L* may exist in an ‘immune-excluded’ TME, consistent with higher stromal scores and activation of TGF-β signaling pathways. Despite the currently unclear role of *TMEM59L* in T cell suppression, our study indicated that *TMEM59L* could represent a potential novel immune target, and the application of anti-*TMEM59L* antibodies after other therapeutic interventions may be an effective therapeutic strategy.

The study bears few limitations. First, the bioinformatic analysis needs to be corroborated by experimental validation *via* immunostaining of the normal and tumor tissues. Then, mechanistic investigation is required to confirm the functional association between *TMEM59L* and cancer- and immune pathways, as well as the epigenetic regulation of *TMEM59L* expression in specific cancers.

In conclusion, by combining a multi-omics approach, we comprehensively explored *TMEM59L* gene expression signature, its prognostic value, as well as its association with immune cell infiltration and cancer-associated pathways in various cancer types. Our findings revealed that *TMEM59L* expression was correlated with poor prognosis across multiple tumor types, especially in COAD, KIRP, and KIRC. Moreover, our study also indicated that *TMEM59L* may represent a potential novel immune target and could play an immune-regulatory role in tumors. This study underscores the importance of *TMEM59L* as a prognostic biomarker and a treatment target and identified an area to be explored further in the future.

## Data availability statement

The datasets presented in this study can be found in online repositories. The names of the repository/repositories and accession number(s) can be found in the article/**Supplementary Material**.

## Author contributions

Conceptualization, CS, and LS; Data curation, analysis and validation, CS, LS, LZ, DC and HW, WQ, PZ, HG; Writing—original draft, CS, and LS; Writing—review and editing, CS, and LS. All authors contributed to the article and approved the submitted version.

## Supplementary material

Figure S1 The expression of TMEM59L in normal and cancer tissues based on the HPA database. (A) The expression of TMEM59L in healthy tissues. (B) The expression of TMEM59L in human cancer tissues. (C) The representative IHC images of TMEM59L in colorectal and renal cancer based on HPA database .(Scale bar: 100 µm).

Figure S2 The differences of EMT pathway activity between high and low TMEM59L mRNA expression in different types of cancer. (A) BRCA, (B) BLCA, (C) COAD, (D) ESCA, (E) READ, (F) OV, (G) TGCT, (H) THCA, (I) STAD.

Figure S3 The single-cell RNA sequencing analysis exhibits the expression pattern as well as the signal pathway of TMEM59L. (A, B) The UMAP projection of all clusters and cell subpopulations. (C, D) TMEM59L expression from BRCA-GSE148673; (E) GSEA showed the the activity of the hallmark EMT pathways in different cells based on TISCH database; (F) GSEA showed the enriched upregulated hallmark pathways in different cells based on TISCH database.

Figure S4 Correlations between TMEM59L and enrichment scores of cancer-associated pathways. (A) Correlations between TMEM59L and the enrichment scores of cancer-associated pathways. (B) Correlations between TMEM59L and the steps of the cancer immunity cycle. Solid lines represent a positive correlation, dashed lines represents a negative correlation, and the the colors represent significant P-values.

Figure S5 The methylation difference between tumor and normal samples of TMEM59L in different human cancers. (A) BLCA, (B) KIRC, (C) KIRP, (D) UCEC, (E) LUSC, (F) LUAD, (G) THCA.

Figure S6 The correlation between methylation and mRNA expression of TMEM59L in different human cancers. (A) BLCA, (B) BRCA, (C) COAD, (D) HNSC, (E) LUAD, (F) PAAD, (G) THCA, (H) UCEC.

## References

[1] Sung H Ferlay J Siegel RL Laversanne M Soerjomataram I Jemal A. Global cancer statistics 2020: GLOBOCAN estimates of incidence and mortality worldwide for 36 cancers in 185 countries. CA: A Cancer J Clin (2021) 71:209–49. doi: 10.3322/caac.21660 33538338

[2] Cao W Chen H Yu Y Li N Chen W. Changing profiles of cancer burden worldwide and in China: a secondary analysis of the global cancer statistics 2020. Chin Med J (Engl) (2021) 134:783–91. doi: 10.1097/CM9.0000000000001474 PMC8104205 33734139

[3] Bray F Laversanne M Weiderpass E Soerjomataram I. The ever-increasing importance of cancer as a leading cause of premature death worldwide. Cancer (2021) 127:3029–30. doi: 10.1002/cncr.33587 34086348

[4] Malvezzi M Carioli G Bertuccio P Negri E La Vecchia C. Relation between mortality trends of cardiovascular diseases and selected cancers in the European union, in 1970–2017. focus on cohort and period effects. Eur J Cancer (2018) 103:341–55. doi: 10.1016/j.ejca.2018.06.018 30029971

[5] Tahmasebi Birgani M Carloni V. Data from: Tumor microenvironment, a paradigm in hepatocellular carcinoma progression and therapy. Int J Mol Sci (2017) 18(2):405. doi: 10.3390/ijms18020405 28216578 PMC5343939

[6] Zhang H Grizzle WE. Exosomes: A novel pathway of local and distant intercellular communication that facilitates the growth and metastasis of neoplastic lesions. Am J Pathol (2014) 184:28–41. doi: 10.1016/j.ajpath.2013.09.027 24269592 PMC3873490

[7] Quail DF Joyce JA. Microenvironmental regulation of tumor progression and metastasis. Nat Med (2013) 19:1423–37. doi: 10.1038/nm.3394 PMC3954707 24202395

[8] Catalano V Turdo A Di Franco S Dieli F Todaro M Stassi G. Tumor and its microenvironment: A synergistic interplay. Semin Cancer Biol (2013) 23:522–32. doi: 10.1016/j.semcancer.2013.08.007 24012661

[9] Roma-Rodrigues C Mendes R Baptista PV Fernandes AR. Data from: Targeting tumor microenvironment for cancer therapy. Int J Mol Sci (2019) 20(4):840. doi: 10.3390/ijms20040840 30781344 PMC6413095

[10] Hanahan D Coussens LM. Accessories to the crime: Functions of cells recruited to the tumor microenvironment. Cancer Cell (2012) 21:309–22. doi: 10.1016/j.ccr.2012.02.022 22439926

[11] Hanahan D Weinberg RA. Hallmarks of cancer: The next generation. Cell (2011) 144:646–74. doi: 10.1016/j.cell.2011.02.013 21376230

[12] Lyssiotis CA Kimmelman AC. Metabolic interactions in the tumor microenvironment. Trends Cell Biol (2017) 27:863–75. doi: 10.1016/j.tcb.2017.06.003 PMC5814137 28734735

[13] Govaert KM Emmink BL Nijkamp MW Cheung ZJ Steller EJA Fatrai S. Hypoxia after liver surgery imposes an aggressive cancer stem cell phenotype on residual tumor cells. Ann Surg (2014) 259(4):750–9. doi: 10.1097/SLA.0b013e318295c160 24253142

[14] Stanta G Bonin S. Overview on clinical relevance of intra-tumor heterogeneity. Front Med (Lausanne) (2018) 5:85. doi: 10.3389/fmed.2018.00085 29682505 PMC5897590

[15] Wang M Zhao J Zhang L Wei F Lian Y Wu Y. Role of tumor microenvironment in tumorigenesis. J Cancer (2017) 8:761–73. doi: 10.7150/jca.17648 PMC5381164 28382138

[16] Assaraf YG Brozovic A Gonçalves AC Jurkovicova D Linē A Machuqueiro M. The multi-factorial nature of clinical multidrug resistance in cancer. Drug Resist Update (2019) 46:100645. doi: 10.1016/j.drup.2019.100645 31585396

[17] Leonetti A Wever B Mazzaschi G Assaraf YG Rolfo C Quaini F. Molecular basis and rationale for combining immune checkpoint inhibitors with chemotherapy in non-small cell lung cancer. Drug Resist Update (2019) 46:100644. doi: 10.1016/j.drup.2019.100644 31585395

[18] Chen Y Tan W Wang C. Tumor-associated macrophage-derived cytokines enhance cancer stem-like characteristics through epithelial-mesenchymal transition. Onco Targets Ther (2018) 11:3817–26. doi: 10.2147/OTT.S168317 PMC6038883 30013362

[19] Colak S Medema JP. Cancer stem cells – important players in tumor therapy resistance. FEBS J (2014) 281:4779–91. doi: 10.1111/febs.13023 25158828

[20] Rycaj K Tang DG. Cancer stem cells and radioresistance. Int J Radiat Biol (2014) 90:615–21. doi: 10.3109/09553002.2014.892227 PMC4341971 24527669

[21] Eppert K Takenaka K Lechman ER Waldron L Nilsson B van Galen P. Stem cell gene expression programs influence clinical outcome in human leukemia. Nat Med (2011) 17:1086–93. doi: 10.1038/nm.2415 21873988

[22] Kim J Joo H Moon H Lee Y. A case of amblyomma testudinarium tick bite in a Korean woman. Korean J Parasitol (2010) 48:313–17. doi: 10.3347/kjp.2010.48.4.313 PMC3018581 21234234

[23] Ben-Porath I Thomson MW Carey VJ Ge R Bell GW Regev A. An embryonic stem cell–like gene expression signature in poorly differentiated aggressive human tumors. Nat Genet (2008) 40:499–507. doi: 10.1038/ng.127 18443585 PMC2912221

[24] Kang G Chen K Yang F Chuai S Zhao H Zhang K. Monitoring of circulating tumor DNA and its aberrant methylation in the surveillance of surgical lung cancer patients: protocol for a prospective observational study. BMC Cancer (2019) 19:579. doi: 10.1186/s12885-019-5751-9 31195991 PMC6567563

[25] Sina AAI Carrascosa LG Liang Z Grewal YS Wardiana A Shiddiky MJA. Epigenetically reprogrammed methylation landscape drives the DNA self-assembly and serves as a universal cancer biomarker. Nat Commun (2018) 9:4915. doi: 10.1038/s41467-018-07214-w 30514834 PMC6279781

[26] Micevic G Theodosakis N Bosenberg M. Aberrant DNA methylation in melanoma: biomarker and therapeutic opportunities. Clin Epigenet (2017) 9:34. doi: 10.1186/s13148-017-0332-8 PMC5381063 28396701

[27] Nakaoka T Saito Y Saito H. Data from: Aberrant DNA methylation as a biomarker and a therapeutic target of cholangiocarcinoma. Int J Mol Sci (2017) 18(6):1111. doi: 10.3390/ijms18061111 28545228 PMC5485935

[28] Elson GCA de Coignac AB Aubry J Delneste Y Magistrelli G Holzwarth J. BSMAP, a novel protein expressed specifically in the brain whose gene is localized on chromosome 19p12. Biochem Biophys Res Commun (1999) 264:55–62. doi: 10.1006/bbrc.1999.1481 10527841

[29] Mannherz O Mertens D Hahn M Lichter P. Functional screening for proapoptotic genes by reverse transfection cell array technology. Genomics (2006) 87:665–72. doi: 10.1016/j.ygeno.2005.12.009 16503394

[30] Ullrich S Münch A Neumann S Kremmer E Tatzelt J Lichtenthaler SF. The novel membrane protein TMEM59 modulates complex glycosylation, cell surface expression, and secretion of the amyloid precursor protein. J Biol Chem (2010) 285:20664–74. doi: 10.1074/jbc.M109.055608 PMC2898310 20427278

[31] Zheng Q Zheng X Zhang L Luo H Qian L Fu X. The neuron-specific protein TMEM59L mediates oxidative stress-induced cell death. Mol Neurobiol (2017) 54:4189–200. doi: 10.1007/s12035-016-9997-9 PMC5288309 27324899

[32] Boada-Romero E Letek M Fleischer A Pallauf K Ramón-Barros C Pimentel-Muiños FX. TMEM59 defines a novel ATG16L1-binding motif that promotes local activation of LC3. EMBO J (2013) 32:566–82. doi: 10.1038/emboj.2013.8 PMC3579146 23376921

[33] Bakulski KM Dolinoy DC Sartor MA Paulson HL Konen JR Lieberman AP. Genome-wide DNA methylation differences between late-onset alzheimer's disease and cognitively normal controls in human frontal cortex. J Alzheimer's Dis (2012) 29:571–88. doi: 10.3233/JAD-2012-111223 PMC3652332 22451312

[34] Hänzelmann S Castelo R Guinney J. GSVA: gene set variation analysis for microarray and RNA-seq data. BMC Bioinf (2013) 14:7. doi: 10.1186/1471-2105-14-7 PMC3618321 23323831

[35] Newman AM Liu CL Green MR Gentles AJ Feng W Xu Y. Robust enumeration of cell subsets from tissue expression profiles. Nat Methods (2015) 12:453–57. doi: 10.1038/nmeth.3337 PMC4739640 25822800

[36] Yoshihara K Shahmoradgoli M Martínez E Vegesna R Kim H Torres-Garcia W. Inferring tumour purity and stromal and immune cell admixture from expression data. Nat Commun (2013) 4:2612. doi: 10.1038/ncomms3612 24113773 PMC3826632

[37] Charoentong P Finotello F Angelova M Mayer C Efremova M Rieder D. Pan-cancer immunogenomic analyses reveal genotype-immunophenotype relationships and predictors of response to checkpoint blockade. Cell Rep (2017) 18:248–62. doi: 10.1016/j.celrep.2016.12.019 28052254

[38] Yang W Soares J Greninger P Edelman EJ Lightfoot H Forbes S. Genomics of drug sensitivity in cancer (GDSC): a resource for therapeutic biomarker discovery in cancer cells. Nucleic Acids Res (2013) 41:D955–61. doi: 10.1093/nar/gks1111 PMC3531057 23180760

[39] Basu A Bodycombe NE Cheah JH Price EV Liu K Schaefer GI. An interactive resource to identify cancer genetic and lineage dependencies targeted by small molecules. Cell (2013) 154:1151–61. doi: 10.1016/j.cell.2013.08.003 PMC3954635 23993102

[40] Niu Z Shi Q Zhang W Shu Y Yang N Chen B. Caspase-1 cleaves PPAR纬 for potentiating the pro-tumor action of TAMs. Nat Commun (2017) 8:766. doi: 10.1038/s41467-017-00523-6 28974683 PMC5626701

[41] Fiaschi T Marini A Giannoni E Taddei ML Gandellini P De Donatis A. Reciprocal metabolic reprogramming through lactate shuttle coordinately influences tumor-stroma interplay. Cancer Res (2012) 72:5130–40. doi: 10.1158/0008-5472.CAN-12-1949 22850421

[42] Giannoni E Bianchini F Masieri L Serni S Torre E Calorini L. Reciprocal activation of prostate cancer cells and cancer-associated fibroblasts stimulates epithelial-mesenchymal transition and cancer stemness. Cancer Res (2010) 70:6945–56. doi: 10.1158/0008-5472.CAN-10-0785 20699369

[43] Hu J Yu A Othmane B Qiu D Li H Li C. Siglec15 shapes a non-inflamed tumor microenvironment and predicts the molecular subtype in bladder cancer. Theranostics (2021) 11:3089–108. doi: 10.7150/thno.53649 PMC7847675 33537076

[44] Chen DS Mellman I. Oncology meets immunology: the cancer-immunity cycle. Immunity (2013) 39:1–10. doi: 10.1016/j.immuni.2013.07.012 23890059

[45] Xu L Deng C Pang B Zhang X Liu W Liao G. TIP: A web server for resolving tumor immunophenotype profiling. Cancer Res (2018) 78:6575–80. doi: 10.1158/0008-5472.CAN-18-0689 30154154

[46] Li B Zhang B Wang X Zeng Z Huang Z Zhang L. Expression signature, prognosis value, and immune characteristics of siglec-15 identified by pan-cancer analysis. Oncoimmunology (2020) 9:1807291. doi: 10.1080/2162402X.2020.1807291 32939323 PMC7480813

[47] Shibue T Weinberg RA. EMT, CSCs, and drug resistance: the mechanistic link and clinical implications. Nat Rev Clin Oncol (2017) 14:611–29. doi: 10.1038/nrclinonc.2017.44 PMC5720366 28397828

[48] Seguin L Desgrosellier JS Weis SM Cheresh DA. Integrins and cancer: regulators of cancer stemness, metastasis, and drug resistance. Trends Cell Biol (2015) 25:234–40. doi: 10.1016/j.tcb.2014.12.006 PMC4380531 25572304

[49] Zhang C Chen T Li Z Liu A Xu Y Gao Y. Depiction of tumor stemlike features and underlying relationships with hazard immune infiltrations based on large prostate cancer cohorts. Brief Bioinform (2021) 22:a211. doi: 10.1093/bib/bbaa211 32856039

[50] Malta TM Sokolov A Gentles AJ Burzykowski T Poisson L Weinstein JN. Machine learning identifies stemness features associated with oncogenic dedifferentiation. Cell (2018) 173:338–54. doi: 10.1016/j.cell.2018.03.034 PMC5902191 29625051

[51] Friedmann-Morvinski D Verma IM. Dedifferentiation and reprogramming: origins of cancer stem cells. EMBO Rep (2014) 15:244–53. doi: 10.1002/embr.201338254 PMC3989690 24531722

[52] Valieris R Amaro L Osório C Bueno AP Rosales MR Carraro DM. Deep learning predicts underlying features on pathology images with therapeutic relevance for breast and gastric cancer. Cancers (Basel) (2020) 12:3687. doi: 10.3390/cancers12123687 33316873 PMC7763049

[53] Moynahan ME Jasin M. Mitotic homologous recombination maintains genomic stability and suppresses tumorigenesis. Nat Rev Mol Cell Biol (2010) 11:196–207. doi: 10.1038/nrm2851 20177395 PMC3261768

[54] Konstantinopoulos PA Waggoner S Vidal GA Mita M Moroney JW Holloway R. Single-arm phases 1 and 2 trial of niraparib in combination with pembrolizumab in patients with recurrent platinum-resistant ovarian carcinoma. JAMA Oncol (2019) 5:1141–49. doi: 10.1001/jamaoncol.2019.1048 PMC6567832 31194228

[55] Hoppe MM Sundar R Tan DSP Jeyasekharan AD. Biomarkers for homologous recombination deficiency in cancer. JNCI: J Natl Cancer Institute (2018) 110:704–13. doi: 10.1093/jnci/djy085 29788099

[56] Heeke AL Baker T Lynce F Pishvaian MJ Isaacs C. Prevalence of homologous recombination deficiency among all tumor types. J Clin Oncol (2017) 35:1502. doi: 10.1200/JCO.2017.35.15_suppl.1502

[57] Sharma P Barlow WE Godwin AK Pathak H Isakova K Williams D. Impact of homologous recombination deficiency biomarkers on outcomes in patients with triple-negative breast cancer treated with adjuvant doxorubicin and cyclophosphamide (SWOG S9313). Ann Oncol (2018) 29:654–60. doi: 10.1093/annonc/mdx821 PMC5888953 29293876

[58] McGranahan N Furness AJ Rosenthal R Ramskov S Lyngaa R Saini SK. Clonal neoantigens elicit T cell immunoreactivity and sensitivity to immune checkpoint blockade. Science (2016) 351:1463–69. doi: 10.1126/science.aaf1490 PMC4984254 26940869

[59] Van Allen EM Miao D Schilling B Shukla SA Blank C Zimmer L. Genomic correlates of response to CTLA-4 blockade in metastatic melanoma. Science (2015) 350:207–11. doi: 10.1126/science.aad0095 PMC5054517 26359337

[60] Rizvi NA Hellmann MD Snyder A Kvistborg P Makarov V Havel JJ. Cancer immunology. mutational landscape determines sensitivity to PD-1 blockade in non-small cell lung cancer. Science (2015) 348:124–28. doi: 10.1126/science.aaa1348 PMC4993154 25765070

[61] Yap TA Gerlinger M Futreal PA Pusztai L Swanton C. Intratumor heterogeneity: seeing the wood for the trees. Sci Transl Med (2012) 4:110p–27p. doi: 10.1126/scitranslmed.3003854 22461637

[62] Yang C Huang X Li Y Chen J Lv Y Dai S. Prognosis and personalized treatment prediction in TP53-mutant hepatocellular carcinoma: an in silico strategy towards precision oncology. Brief Bioinform (2021) 22:a164. doi: 10.1093/bib/bbaa164 32789496

[63] Zhang S Dai H Li W Wang R Wu H Shen M. TMEM116 is required for lung cancer cell motility and metastasis through PDK1 signaling pathway. Cell Death Dis (2021) 12:1086. doi: 10.1038/s41419-021-04369-1 34789718 PMC8599864

[64] Schmit K Michiels C. TMEM proteins in cancer: A review. Front Pharmacol (2018) 9:1345. doi: 10.3389/fphar.2018.01345 30574087 PMC6291505

[65] Li B Huang MZ Wang XQ Tao BB Zhong J Wang XH. Erratum: TMEM140 is associated with the prognosis of glioma by promoting cell viability and invasion. J Hematol Oncol (2015) 8:101. doi: 10.1186/s13045-015-0199-0 26329470 PMC4556009

[66] Marx S Dal Maso T Chen J Bury M Wouters J Michiels C. Transmembrane (TMEM) protein family members: Poorly characterized even if essential for the metastatic process. Semin Cancer Biol (2020) 60:96–106. doi: 10.1016/j.semcancer.2019.08.018 31454669

[67] Salmon H Remark R Gnjatic S Merad M. Host tissue determinants of tumour immunity. Nat Rev Cancer (2019) 19:215–27. doi: 10.1038/s41568-019-0125-9 PMC7787168 30867580

[68] Cogdill AP Andrews MC Wargo JA. Hallmarks of response to immune checkpoint blockade. Br J Cancer (2017) 117:1–07. doi: 10.1038/bjc.2017.136 28524159 PMC5520201

[69] Pan C Liu H Robins E Song W Liu D Li Z. Next-generation immuno-oncology agents: current momentum shifts in cancer immunotherapy. J Hematol Oncol (2020) 13:29. doi: 10.1186/s13045-020-00862-w 32245497 PMC7119170

[70] Hamid O Robert C Daud A Hodi FS Hwu WJ Kefford R. Five-year survival outcomes for patients with advanced melanoma treated with pembrolizumab in KEYNOTE-001. Ann Oncol (2019) 30:582–88. doi: 10.1093/annonc/mdz011 PMC6503622 30715153

