# Supplementary figures and images for: Prognostic value of TMEM59L and its genomic and immunological characteristics in cancer
Source: Front Immunol. 2022 Dec 23;13:1054157. doi: 10.3389/fimmu.2022.1054157 (PMC9816415; doi:10.3389/fimmu.2022.1054157)

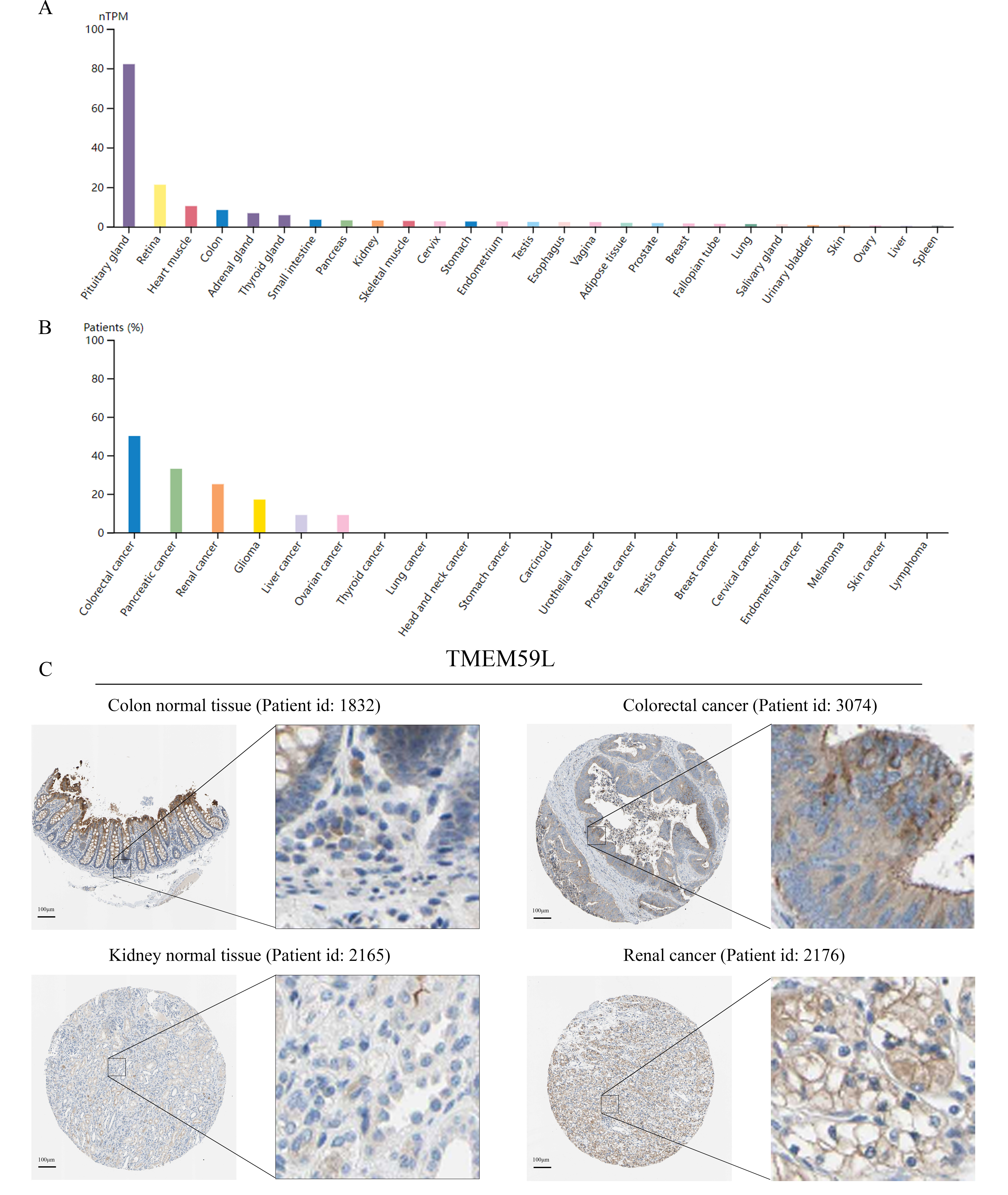

Supplement: Figure S1 — The expression of TMEM59L in normal and cancer tissues based on the HPA database. (A) The expression of TMEM59L in healthy tissues. (B) The expression of TMEM59L in human cancer tissues. (C) The representative IHC images of TMEM59L in colorectal and renal cancer based on HPA database .(Scale bar: 100 µm). [file Image_1.jpeg]

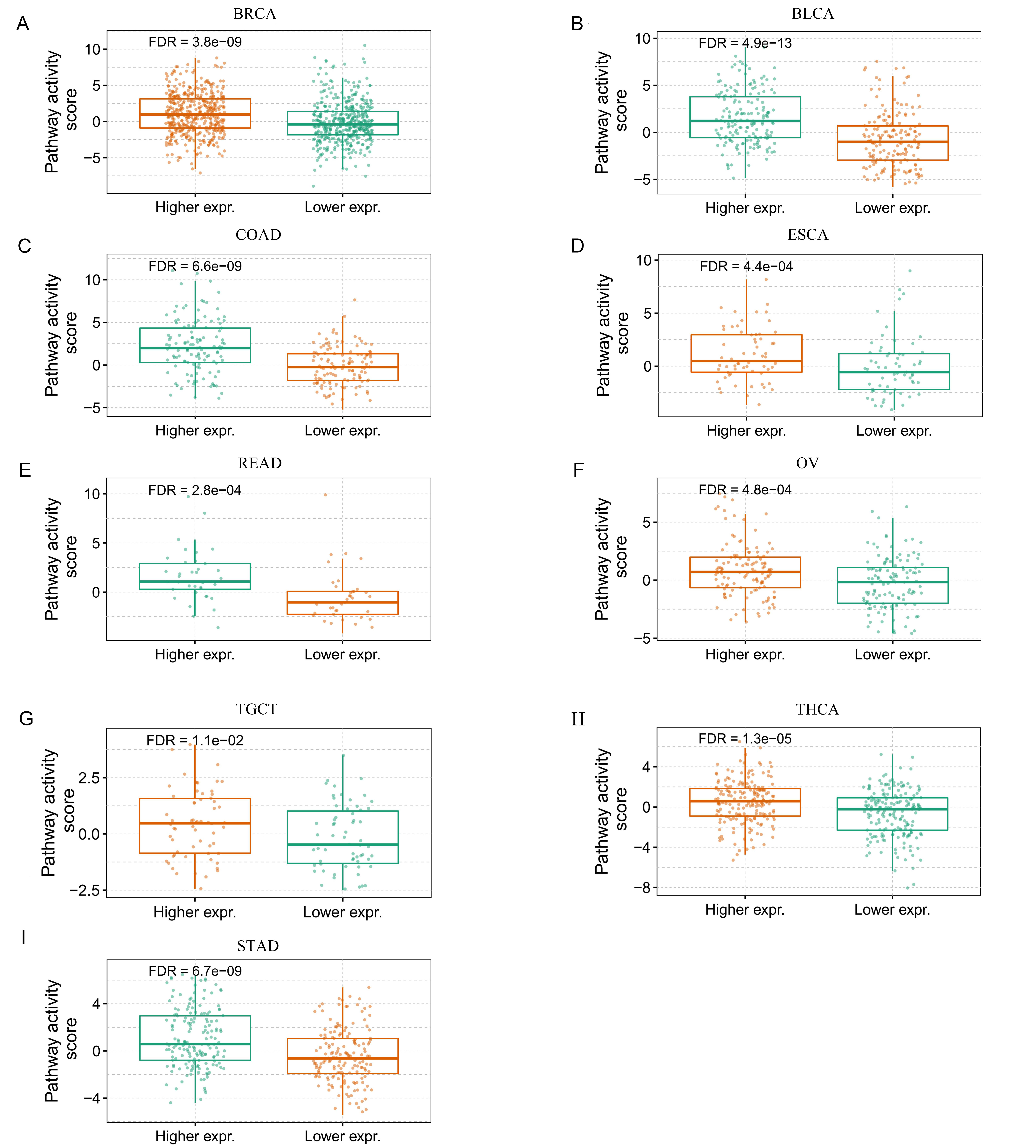

Supplement: Figure S2 — The differences of EMT pathway activity between high and low TMEM59L mRNA expression in different types of cancer. (A) BRCA, (B) BLCA, (C) COAD, (D) ESCA, (E) READ, (F) OV, (G) TGCT, (H) THCA, (I) STAD. [file Image_2.jpeg]

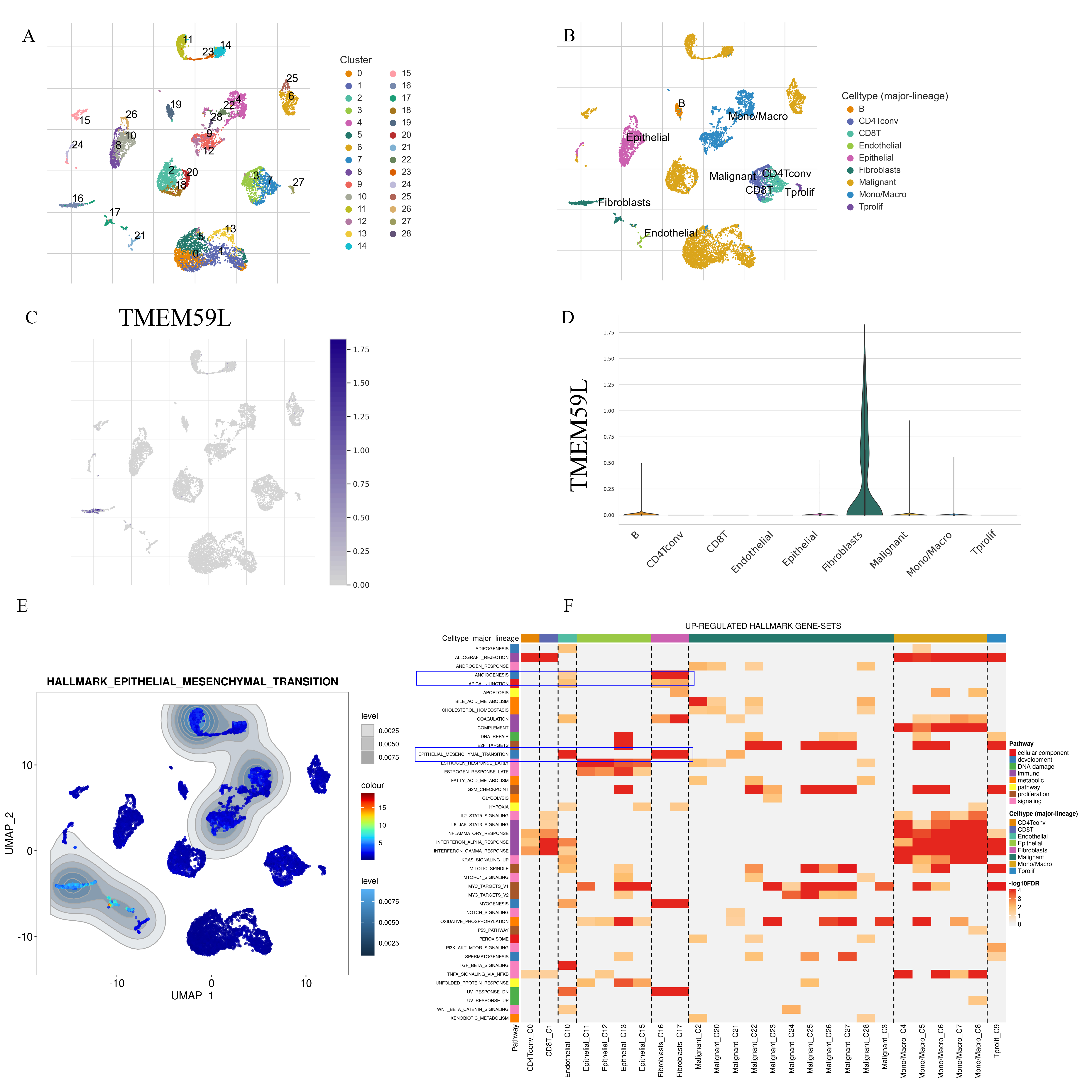

Supplement: Figure S3 — The single-cell RNA sequencing analysis exhibits the expression pattern as well as the signal pathway of TMEM59L. (A, B) The UMAP projection of all clusters and cell subpopulations. (C, D) TMEM59L expression from BRCA-GSE148673; (E) GSEA showed the the activity of the hallmark EMT pathways in different cells based on TISCH database; (F) GSEA showed the enriched upregulated hallmark pathways in different cells based on TISCH database. [file Image_3.jpeg]

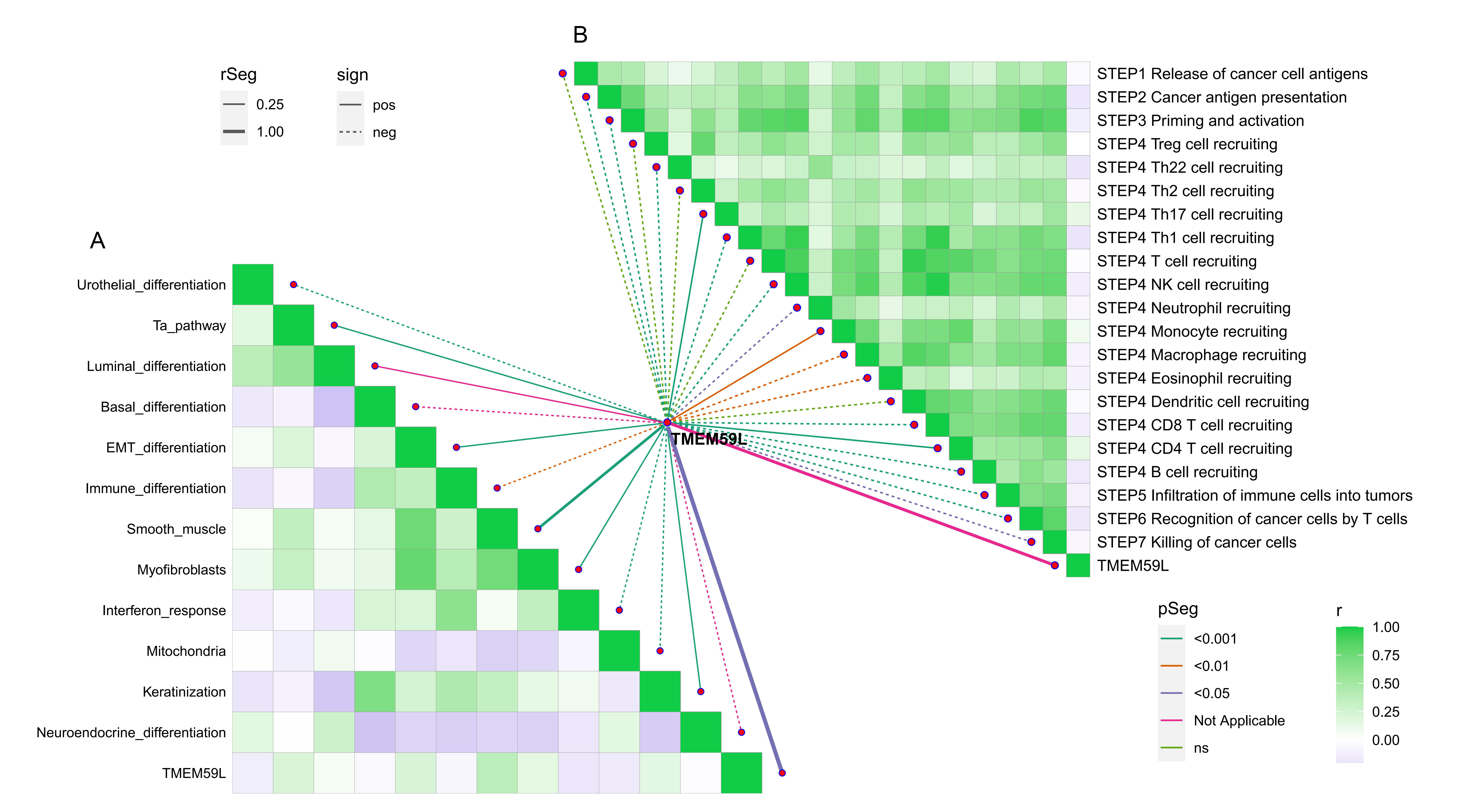

Supplement: Figure S4 — Correlations between TMEM59L and enrichment scores of cancer-associated pathways. (A) Correlations between TMEM59L and the enrichment scores of cancer-associated pathways. (B) Correlations between TMEM59L and the steps of the cancer immunity cycle. Solid lines represent a positive correlation, dashed lines represents a negative correlation, and the the colors represent significant P-values. [file Image_4.jpeg]

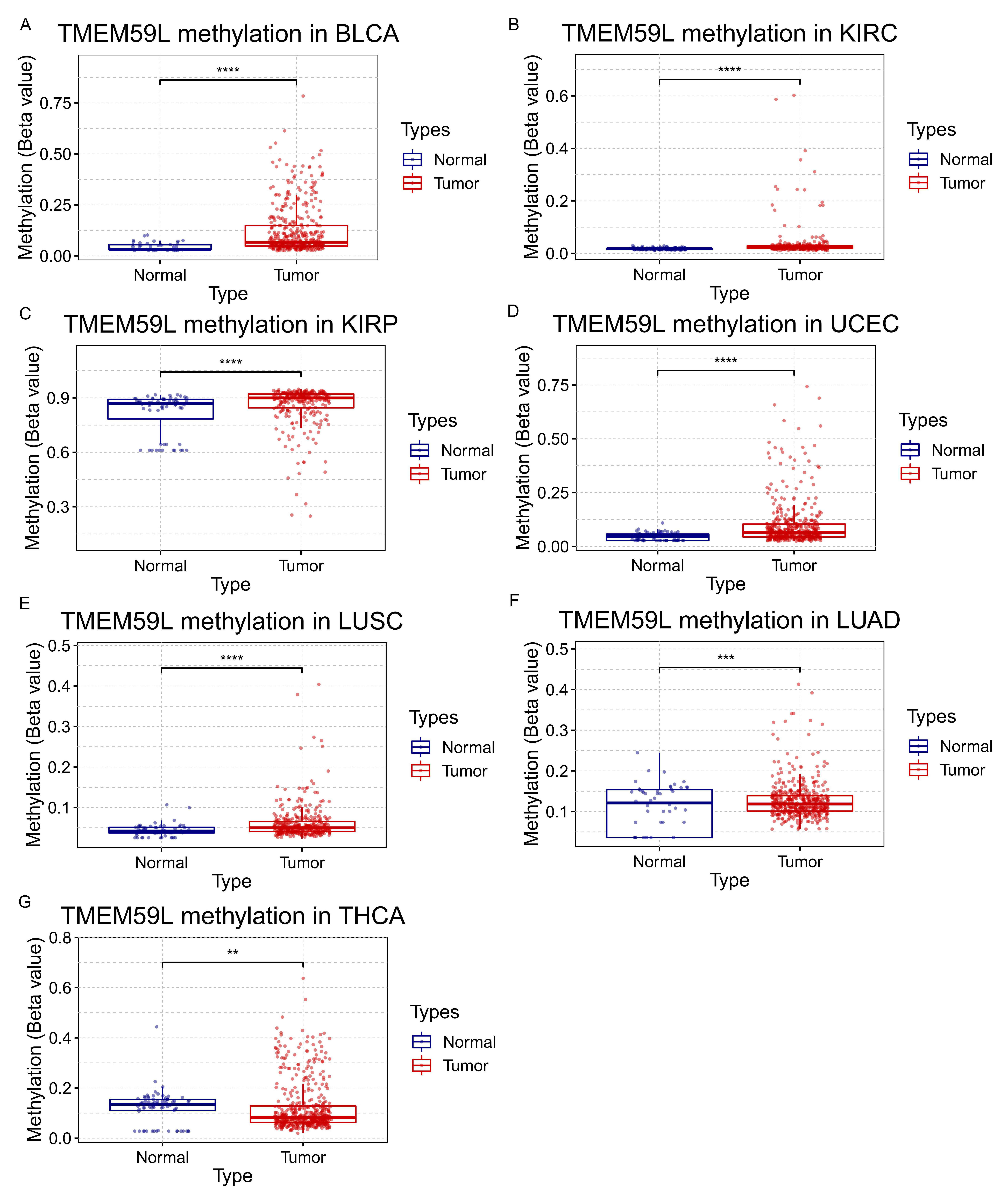

Supplement: Figure S5 — The methylation difference between tumor and normal samples of TMEM59L in different human cancers. (A) BLCA, (B) KIRC, (C) KIRP, (D) UCEC, (E) LUSC, (F) LUAD, (G) THCA. [file Image_5.jpeg]

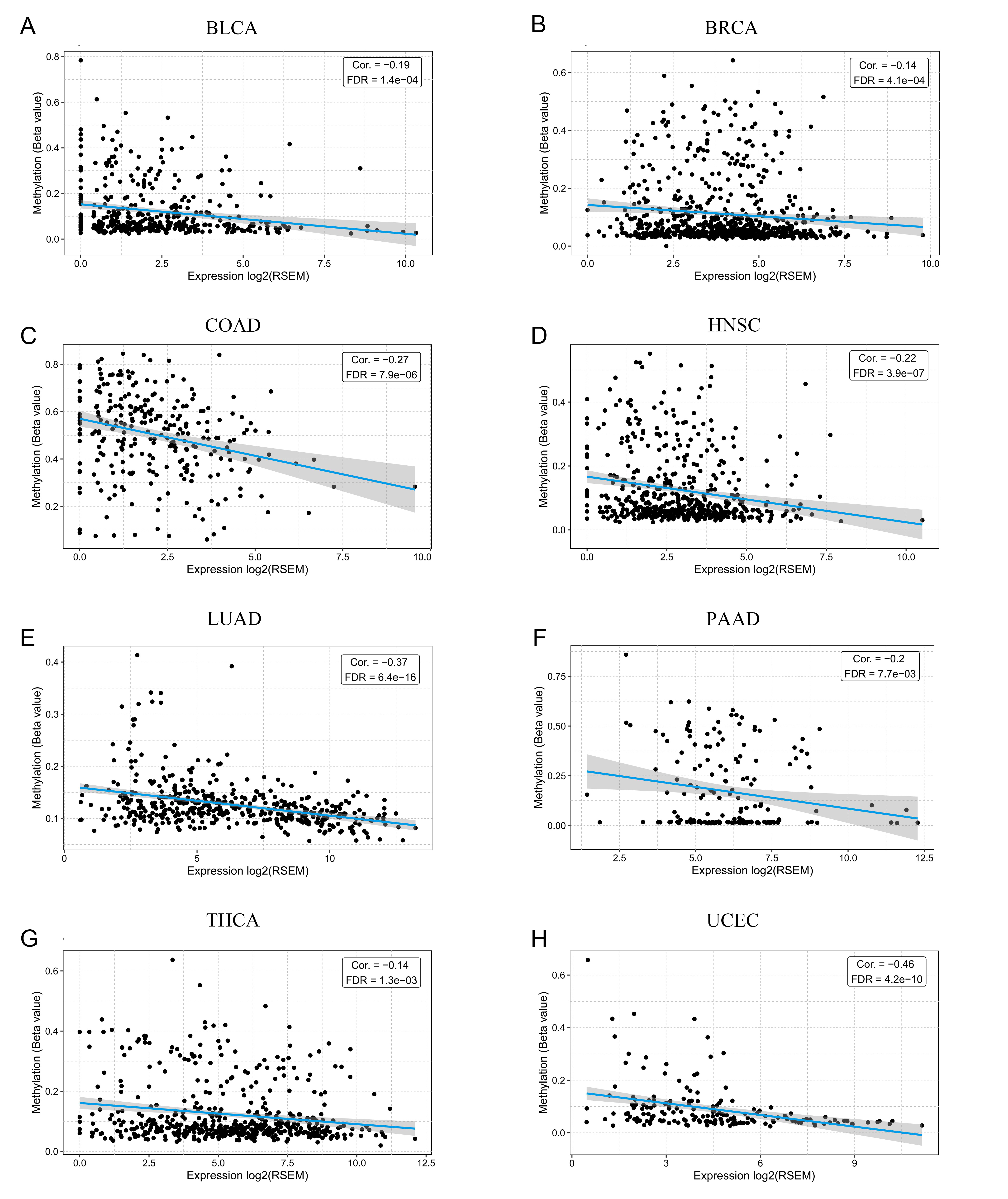

Supplement: Figure S6 — The correlation between methylation and mRNA expression of TMEM59L in different human cancers. (A) BLCA, (B) BRCA, (C) COAD, (D) HNSC, (E) LUAD, (F) PAAD, (G) THCA, (H) UCEC. [file Image_6.jpeg]
